# (Global) single market for data? Exploring data cosmopolitanism as a solidarity-based regulatory approach to the european health data space

**DOI:** 10.1016/j.dib.2026.113016

**Published:** 2026-06-22

**Authors:** Marta Musidlowska, Michiel Fierens, Isabela Maria Rosal

**Affiliations:** a KULeuven, Centre for IT and IP Law, Leuven, Belgium; b KULeuven, imec, Centre for IT and IP Law, Leuven, Belgium

**Keywords:** Internal market, Global data spaces, Data space, European health data space, Data solidarity, Data cosmopolitanism

## Abstract

The European Union (EU) is advancing an interoperable data governance framework through the establishment of Common European Data Spaces (CEDS), fostering innovation, economic competitiveness, and the provision of personalised services. Beyond these goals, CEDS seem to reflect ambitions of digital sovereignty and strategic autonomy in the global data economy, aiming to position itself among global leaders in Artificial Intelligence (AI) development and innovation. This article critically examines these ambitions through the recently adopted European Health Data Space Regulation (EHDSR), a flagship initiative establishing common mechanisms that enables secondary use of health data for research, innovation, and public health responses. The analysis highlights tensions between the EU’s pursuit of autonomy and its foundational principles of solidarity and international cooperation, especially regarding engagement with non-EU countries. While Article 114 of the Treaty on the Functioning of the EU (TFEU) underpins its current data space legislation, it limits the EU’s capacity to achieve broader geopolitical objectives. The paper advances the concept of “data cosmopolitanism” as an extension of the solidarity principle, proposing a theoretical framework for a global approach to health data sharing that incorporates societal values such as social justice and equality in access to healthcare.

## 1 Introduction

The EU is implementing a series of substantial legislative initiatives with the objective of streamlining the process of information sharing within the European internal market. The aim is to impose an integrated EU data governance regime [1]. As outlined in the EU Data Strategy, the establishment of sectoral CEDS is intended to shift the focus from mere data collection to data innovation, thereby facilitating the emergence of new, personalised services [2]. The core concept of CEDS can be summarised as the tangible implementation of a novel EU data governance regime. This regime is delineated by a governance framework and underlying technical infrastructure, which are aligned with specific design principles. Although this will be an incremental evolution, with no ready-made, standard model of a CEDS, this paper builds on some hypotheses regarding the incremental evolution of the governance and technical components.

The EU has demonstrated an ambition to take up a leading role in the formation of global standards and an environment that supports data-driven solutions alongside technological development. Despite the primary narrative focus on the usefulness of data aimed at improving the quality of services or products, the creation of the European internal market for data has not only an economic, but also a strategic, dimension in the geopolitical context. The perception of data as a strategic asset, along with the ambition to foster the creation of a data-driven society and economy, has been reflected in over a decade of EU policies and regulatory frameworks [2]. Most recently, overcoming data silos and improving access to data within and across strategic sectors have been seen as prerequisites for the development of AI and for strengthening the EU’s position in the global innovation race, led by the United States (US) and China [3]. In this context, data and governance can also be viewed as a key pillar of the EU's “strategic autonomy” and “digital (or data) sovereignty” - terms commonly used in policy documents that address the challenge of the EU’s economic competitiveness in the global data economy [4], especially in the context of recent ambition to turn the EU into an “AI Continent” [3].

The notion of economic sovereignty and autonomy through a European single market is frequently invoked by EU policies to substantiate strategies aimed at reducing the EU's reliance on external jurisdictions when sharing data. Moreover, the EU's approach to data governance is proposed as a means to ensure compliance with human rights principles. In the event that third countries other than those within the European Union wish to share data within the EU market, they are required to comply with the associated regulations. Furthermore, CEDS are subject to technical requirements and standards that are not immediately apparent. Consequently, third countries may encounter challenges in adhering to these requirements, which may give rise to undisclosed security objectives. The concepts of sovereignty and autonomy are therefore subject to debate, as they are intertwined by divergent, at times incongruent, and at other times collaborative objectives. In that context, the present paper aims to suggest another lens through which the regulation of data governance in the health sector might be approached to. More specifically, this paper critically examines the European Union’s reliance on Article 114 TFEU as the primary legal basis for data governance initiatives, arguing that its internal market focus may be ill-suited to address the broader geopolitical, global, and solidarity-oriented dimensions of health data governance. However, these policy instruments do not tackle solely internal single market matters, which goes beyond the scope of the legal basis. It is in response to these limitations that the paper proposes an alternative perspective.

Within the context of the strategic and geopolitical pillar, the Data Union strategy addresses the participation of non-EU countries in the data sharing ecosystem, emphasising the EU's current excessive reliance on other countries for access to data relevant for developing generative AI [5]. Consequently, the EU is implementing additional measures to ensure the secure export of data, while concurrently fostering the import of data from non-EU countries. Conversely, within the formulation of policies pertaining to CEDS, underlying narratives of strategic autonomy or digital sovereignty are often referred to as "hybrid," giving rise to both classic normative powers for developing an Internal Market, as well as geopolitical aspirations and protectionist measures [4]. In other words, hybrid policies are understood as instruments that aim at more than one objective, not necessarily or clearly related. Often, not all objectives are clearly outlined in these policies, posing a need for a further and contextual exploration of the legal instruments.

This prompts a crucial question: In the context of global collaboration, particularly with non-EU countries, how can policy objectives, centred on autonomy, shaped by the practical and geopolitical context, be reconciled with the ideals of other foundational European values, such as the principle of solidarity, as enshrined in Article 21 of the TFEU? Furthermore, in the context of Articles 179 and 180 TFEU, cooperation with third countries is explicitly identified as a means of enhancing the EU's scientific and technological foundations. This is particularly relevant in the context of the Data Union Strategy, which aims to enhance collaboration in data exchange between the EU and third countries, while reaffirming the EU's geopolitical digital sovereignty (economically and in terms of security) in light of the current practical geopolitical situation. Interestingly, these divergent interests appear to be in direct opposition to the established ideals of the European Union and, by extension, other European values. Such tensions give rise to the question of whether the Union is both authorised and adequately equipped to design and implement hybrid policies to shape CEDS in the way they currently do, or if they ought to be approached from a different point of view to balance out with other European values such as solidarity.

In this context, the adoption of EHDSR can be perceived as one of the key achievements towards the development of CEDS in strategic areas [6,7]. The global pandemic of 2020 highlighted the necessity for establishing a common framework for the secondary use of electronic health data, enabling, inter alia, prompt access by public sector bodies to address public health crises, as well as improve research and innovation activities in healthcare, including training algorithms in medical devices or AI systems. While the EHDSR is, at least at the level of formal objectives, presented as an instrument to enhance the quality, efficiency, and accessibility of healthcare, particularly through a solidarity-based framework for the secondary use of health data (see, inter alia, [8]), such a characterisation only partially reflects its broader significance. Beyond these normative commitments, the initiative may also be understood as embedded within a wider geopolitical and techno-economic context, in which data governance assumes strategic importance. From this perspective, the EHDSR can be interpreted as contributing to the European Union’s efforts to consolidate its position in global technological competition, notably in relation to the development and deployment of artificial intelligence systems, thereby revealing a less explicit, yet structurally significant, policy rationale. These aspects attract criticism in the pursuit of those ideal European values (see, inter alia, [9]).

In this regard, the article critically examines how the EU’s ambitions of strategic autonomy and digital sovereignty are embedded in the legal, governance and technical design of the EHDS, and how these ambitions conflict with other fundamental EU principles, such as solidarity and international cooperation, particularly in the context of healthcare. The analysis does not primarily focus on the political context, nor does it employ empirical methods; rather, it adopts a doctrinal and normative legal approach aimed at reflecting on alternative normative foundations for regulating data infrastructures, thereby opening the debate within legal, policy, and academic contexts. Furthermore, given that numerous CEDS are subject to an incremental evolution and the governance and technical frameworks still require maturation, the EHDS functions as a valuable example for the evaluation of hybrid policies and implicit interests, including economic sovereignty, geopolitics and security, as outlined in the Data Union Strategy. These policies and interests may face challenges when considered in light of the ideals of the EHDS, which is based on other European values such as data solidarity and public health.

Current challenges stem from the EU’s reliance on Article 114 TFEU, a legal basis designed to support the establishment and functioning of the internal market. When EU policymakers move beyond harmonisation to pursue broader objectives—such as data sovereignty, security, and geopolitical positioning—this legal basis may prove structurally insufficient [10]. As a result, the possibility for non-EU countries to join or fully participate in EU data-sharing initiatives is constrained, since Article 114 only supports measures with a direct link to internal market functioning and cannot readily address wider geopolitical objectives or third-country inclusion (see, inter alia, [11]).

In response to these structural limitations, this paper advances the hypothesis of “data cosmopolitanism” as an alternative legal and normative lens for the regulation and further evolution of “framework for multi-country secondary use” with consideration of the global interests (Recital 80 EHDSR). This approach allows for the internationalisation of the concept of data solidarity, that normally focuses on the commitment “to equitably distribute the benefits and burdens associated with data initiatives in a population” [12,13]. In the context of EHDS, data cosmopolitanism can be explored through the potential participation of non-EU countries in a health data space that brings benefits to the global community, not just the EU. The central claim is that data cosmopolitanism can serve as a theoretical lens through which the legal foundations for internal and external forms of collaboration can be explored.

In this context, the EU’s reliance on Article 114 TFEU as the primary legal basis for data governance initiatives raises important limitations. Designed to support the establishment and functioning of the internal market, Article 114 may be ill-suited to accommodate broader objectives related to global cooperation, public health, and solidarity. This has prompted consideration of alternative legal bases within the Treaties. For instance, Article 352 TFEU allows for more flexible action where necessary to attain Treaty objectives in the absence of specific competences, while Article 216 TFEU enables the Union to conclude agreements with third countries, thereby supporting external dimensions of data governance. Against this background, the present paper explores whether such alternative legal foundations could better accommodate a more globally oriented approach to health data governance, as conceptualised through the lens of data cosmopolitanism.

## 2 Roadmap and Methodology

Part 3 of this paper employs a descriptive methodology, applying the EHDSR as an example. Its findings provide a foundation for proposing an alternative approach to pursuing the ideals underlying the health data-sharing ecosystem and incorporating related European values. This approach is characterised by the concepts of data solidarity and data cosmopolitanism. To develop this argument, the following steps are taken: (i) examination of the legal basis under Article 114 TFEU (Section 3.1), followed by (ii) an analysis of digital sovereignty and strategic autonomy (Section 3.2), (iii) an assessment of the balance between internal and external solidarity (Section 3.3), and finalised with (iv) conclusions regarding the implications for the scope of Article 114 TFEU (Section 3.4).

Building on concepts and understandings from international law, Section 3.1 analyses and critically examines the regulation of the internal market through Article 114 TFEU. This focus is justified by the fact that current data space–related legislation, such as the EHDSR, is underpinned by Article 114 TFEU. As Broeders, Cristiano and Kaminska [4] argue, the implementation of hybrid policies that address both strategic sovereignty objectives under the guise of the internal market, and internal market objectives itself as outlined in Article 114, could potentially exceed the intended scope and application of this article. To understand this assumption, the ensuing sections will provide a detailed analysis of the way the EU has progressively adopted geopolitical and digital sovereignty ambitions under the auspices of Article 114. Furthermore, the notion of solidarity, which should serve as a fundamental guiding principle for EU actions in the international scene, will be examined in relation to the EU's recent endeavours within the context of the internal market. Examining the different dimensions of solidarity helps us to determine whether and how the EU is exceeding its internal market competences and concealing broader sovereignty ambitions under the guise of Article 114 TFEU.

Section 3.2 then concentrates on the implementation of digital sovereignty and strategic autonomy as the prevailing principles guiding the development of CEDS under the EU regulatory regime. This development primarily serves economic, security, and geopolitical objectives, while key European values, such as solidarity and global participation in health data sharing remain in the background. This Section is based on a descriptive and analytical study of relevant EU policy documents, alongside desk research on related legal literature. It also examines how digital sovereignty ambitions, and the instruments designed to achieve these objectives, are implemented in practice through the technical and governance framework of CEDS.

Section 3.3 provides a thorough reflection on the EHDS and the EU Data Strategy, revealing that the EU primarily prioritises internal solidarity rather than accommodating external solidarity approaches, generally understood as EU’s partnership and cooperation with third countries [14].

As external solidarity aligns more closely with the pursuit of a global benefits-oriented framework, Section 3.4. concludes Part 3 by building on the previous findings and demonstrating how, in practice, the EU is extending the remit of Article 114 TFEU in light of its digital sovereignty objectives.

After this analysis, Part 4 presents a normative, hypothesis-driven argument. It develops a theoretical framework that outlines a paradigm for the evolution of CEDS and proposes an alternative approach to the current model. Data cosmopolitanism is introduced as a means of enhancing the global dimension of health data sharing, grounded in the European value of solidarity. This approach allows Member States to uphold national traditions in health data sharing while complying with their international human rights obligations (Section 4.1).

Finally, Part 4 concludes with normative reflections for future research, indicating potential alternative residual legal bases to Article 114 TFEU. It argues that, in the light of data cosmopolitanism, this legal basis could offer a more balanced and transparent way to reconcile the competing objectives that characterize the hybrid and ambiguous policies associated with the EU Data Strategy (Section 4.2).

## 3 (Beyond) European Internal Market: art. 114 TFEU as a Basis for Extending EU Competences

### 3.1 Internal market through 114 TFEU

The TFEU provides several legal bases that can be invoked for the exercise of legislative powers at the EU level, thereby justifying the adoption of Directives, Regulations, and other legal instruments. One such basis is Article 114 TFEU, which empowers EU legislators to adopt measures harmonising national laws and regulations insofar as their objective concerns the establishment and functioning of the internal market. The harmonisation of laws constitutes a shared competence and seeks to address divergences between national legal systems - divergences that are often regarded as among the obstacles to achieving European integration and strengthening the Union’s external influence [15].

Following the TFEU’s definition, internal market^1^ shall be understood as “an area without internal frontiers in which the free movement of goods, persons, services and capital is ensured.” (Article 26(2) TFEU). Nevertheless, free movement of persons is excluded from the scope of Art. 114, showcasing the provision’s focus on free movement of goods [15]. The concept of “internal market” is, nevertheless, broadly interpreted in the application of Article 114 for the approximation of laws, allowing for the adoption of different legal instruments if they intend to improve the functioning of the internal market [16].

Procedural rules also facilitate the use of Article 114 as a legal ground. Acts based on Article 114 must follow the ordinary legislative procedure, allowing for any measure to be adopted by this procedure, such as Directives, but also Regulations. Also, only a qualified majority from the European Council and the Parliament is needed. On contrary, other legal bases have more complex procedures and requirements in place for their application. The adoption of this simplified procedure was followed by a fear from Member States of losing their sovereignty. This, in turn, led to the adoption of the opt-out clauses, allowing for Member States to adopt national instruments contrary to the EU measure. Nevertheless, these opt-out mechanisms must follow specific requirements and procedure, limiting their use [15].

In pursuit of the harmonisation of the EU internal market, legislators relied upon Article 114 as the legal foundation for the adoption of several digital regulations. For instance, the EHDSR, the Data Governance Act (DGA), and the Data Act (DA), are examples of legal instruments adopted by the EU under the Digital Agenda that use Article 114 as a legal basis for their adoption. This trend is further evidenced by the call for evidence on the Digital Omnibus (Digital Package on Simplification), which also references Article 114 TFEU as a legal basis [17,18].

There is an increasing tendency of the EU legislator to rely on Article 114 to regulate digital matters. However, there is compelling evidence that several of those digital matters are initially embedded within the EU's internal market ambition but nonetheless regulate subjects that extend beyond the notion of harmonising and fortifying the market. Also on the matter, the Brussels effect, as defined by Bradford [19], denotes the establishment of regulatory frameworks with the objective of safeguarding technological sovereignty and the enforcement of the EU's fundamental rights approach on the global stage. The concept of the 'Brussels effect' has been proposed as a strategy to safeguard the EU's position in the region's market and mitigate the impact of international influences. However, the choice of Article 114 TFEU as the legal basis for new digital policy instruments places greater emphasis on the EU’s economic, security, and geopolitical objectives, often at the expense of solidarity and international participation. This is because Article 114 TFEU is inherently limited to the internal market. As a result, the development of such instruments may gradually move away from a globally oriented, benefit-sharing approach to health data governance. At the same time, this trajectory may still preserve national traditions, maintain European solidarity-based values of cooperation, and promote a more balanced accommodation of divergent interests within the EHDS.

### 3.2 Digital sovereignty under EU data strategy – unintended consequences?

Digital sovereignty is an expansive concept, encompassing a broad spectrum of areas and subjects. The discourse encompasses not only data (personal and industrial) but also software (e.g., Microsoft Office), standards (cybersecurity and AI certification), hardware, and infrastructure (chips and data centres) [20]. Furthermore, how digital sovereignty and, consequently, autonomy, in terms of influencing the digital sphere, can be achieved are also diverse. In this regard, digital sovereignty is closely aligned with economic, security and, more broadly, geopolitical objectives. For instance, in recent years, the EU has exhibited unmistakable commitment and strategic direction in the realm of digital sovereignty, evidenced by the establishment of specific objectives and the implementation of targeted policies concerning hardware (e.g., the European Chips Act) to achieve digital sovereignty [21].

At the EU level itself, concerns have been frequently raised about the influence of so-called Big Tech international companies on the European digital market. As the European Commission have repeatedly emphasised, Europe must take action to ensure its autonomy by reducing its technological dependencies on the rest of the world and by influencing the digital sphere once more by defining European rules and values for the European internal market [2]. The concept of digital sovereignty has thus become increasingly salient for the general public in recent years as private companies based in other countries, like the US and China, exert influence over a wide range of digital matters. These include the use of US software such as Windows from Microsoft in our daily lives or Google Workspace in schools, or the reliance on Chinese hardware in our telecom networks, and even the use of third-country cloud networks to store our corporate data. In this regard, the EU has expressed its intention to establish itself as a global norm setter in data governance of (industrial) data [22]. As will be demonstrated in the following subsections, these ambitions can be translated into concrete areas specifically tackled by the European Data Strategy and Data Union Strategy, namely, data localisation, standard setting and interoperability, as well as infrastructural ambitions.

#### 3.2.1 Examples of current digital sovereignty regulatory strategies under 114 TFEU

##### 3.2.1.1 Data localisation

Firstly, a more thorough examination of the DGA and the DA discloses a strong emphasis on geographical influence over data storage and thus data localisation [23]. Upon initial examination of the DGA, the EU sought to address the reluctance to share data and the concomitant absence of trust between data holders and potential data users by establishing neutral parties between the parties that share data, namely data intermediaries. These data intermediaries are subject to stringent obligations regarding the further use of data. The services provided are confined to the sharing of data and the facilitation of its subsequent reuse. This is to circumvent the 'vertical integration' of services, as evidenced by the prevailing practice of large platforms within the contemporary data market [24]. However, obligations incumbent on data intermediaries are also measures to enhance data localisation in the EU and its Member States. This approach could be regarded as a strategic manoeuvre to place geopolitical and sovereignty ambitions, covered under the security and economic objectives narrative for keeping the data within the internal market. For instance, as outlined in Article 31 DGA, data intermediaries are obligated to implement all reasonable technical, legal and organisational measures, including contractual provisions, with the aim of preventing international data transfers or governmental access to non-personal data stored within the Union. This is to be implemented in instances where such transfers or access would result in a conflict with Union law or the national legislation of the relevant Member State.

With regard Article 32 DA, international data transfers or requests to access data can be similarly limited by data processing services (broadly speaking cloud services), as those requests need to be considered proportionate, legitimate, and compliant with EU law. In this respect, data stored on data processing services can be regarded as encompassing a wide range of potential data from diverse industrial domains. Once more, allowance is made for those 'European' data processing services to impose restrictions on third countries or parties thereof regarding access to industrial data [2,24]. Both provisions indicate an EU objective to confine data within its territory, thereby limiting the realisation of its potential to the benefit of the Union. In that context, once again, Article 114 TFEU serves as a legal basis to pursue digital sovereignty from the perspective of achieving specific economic and security-oriented goals, illustrating a broader trend in EU digital regulation of relying on internal market competences to advance sovereignty-oriented objectives.

##### 3.2.1.2 Imposing interoperable standards and development of EU-infrastructure

Secondly, the European Data Strategy, encompassing instruments such as the DA and the EHDSR, underscores the EU's ambition to establish its own technical standards for data sharing and data governance. The DA stipulates that participants in CEDS, including the EHDS, are obligated to adhere to essential requirements. This ensures the interoperability and compliance with specific standards. The objective of establishing own standards and promoting interoperability is logical when considered in relation to another objective of the European Data Strategy, namely the development of European or national technology and infrastructure, with the requirement for other providers of technology and infrastructure from outside the EU to comply. The development of a comprehensive data infrastructure is a pivotal component of the initiative [25]. In this regard, a substantial financial commitment of at least 2 billion euros was designated for a European High Impact Project, with the objective of developing data processing infrastructures, data sharing tools, architectures and governance mechanisms to facilitate effective data sharing within CEDS. The project concentrated on the federation of energy-efficient and trustworthy cloud infrastructures and associated services. The establishment of a pan-European cloud services marketplace, encompassing the full spectrum of cloud service offerings, was identified as a significant overarching ambition [2]. Moreover, a considerable financial investment was made in the establishment of the data spaces support centre and in sector-specific follow-up projects [26]. A total of 68 projects is currently being managed in relation to the implementation of a health data space, with a total funding allocation in excess of 100 million [27].

The recent Digital Omnibus proposal also reinforces the need for EU wide standards by, for example, explicitly increasing the topics for which European standardisation organisations should draft standards and providing news attributions related to EU standards to the European Data Innovation Board [18]. The EU's ambition to develop its own approach to data governance and data sharing also encompasses the development of European infrastructure and technology. This is a particularly important consideration within the context of CEDS. The DA delineates the concept of CEDS as purpose- or sector-specific, or cross-sectoral interoperable frameworks for the establishment of common standards and practices for the sharing or joint processing of data for the development of new products and services, scientific research, or civil society initiatives (Article 33 DA). While CEDS was originally conceived with the aim of achieving a strategy of coexistence resulting in a common understanding of data instead of a common infrastructure, there has been a growing trend of the EU establishing more centralised support platforms [28]. In that regard, the implementation of additional technical features is deemed necessary to facilitate the integration of CEDS, thus enabling genuine collaborative use within the internal market [29].

In the light of the above, both examples (data localisation and imposing interoperable standards) illustrate a broader pattern in EU data governance policy frameworks, whereby regulatory and technical frameworks prioritise internal integration within the Union, while conditioning or limiting external participation. This dynamic reinforces a form of internal solidarity but raises questions about the extent to which external or global solidarity can be realised in practice.

### 3.3 Solidarity approaches under EU data strategy and EHDSR

Strategic autonomy and digital sovereignty policies are primarily driven by geopolitical and economic objectives. However, these objectives are embedded within a broader EU data governance framework that simultaneously pursues other aims, including fundamental rights protection, public health, and cross-border cooperation. This results in inherent tensions between competing policy objectives, particularly where economically and geopolitically driven measures interact with value-based commitments.

In this context, such policies often overlook the borderless nature of the digital environment, where traditional national boundaries are increasingly blurred. This creates a normative gap: while the EU promotes integration and data sharing within the internal market, less attention is given to how these frameworks accommodate broader, outward-looking forms of cooperation. In areas such as environmental protection and healthcare, where collective action is essential, a more solidaristic approach to data sharing should not merely be desirable, but normatively necessary to ensure that governance frameworks align with broader European values.

The concept of ‘solidarity’ remains relatively indeterminate, although widely discussed in the social sciences (see, inter alia, [30]). It is frequently invoked in public discourse to justify actions yet often lacks clear operationalisation at the policy level [31]. Within EU law and policy, solidarity has been most extensively developed in areas such as migration, environmental protection, and healthcare, where it functions as a principle that supports collective responsibility and burden-sharing [32].

Against this background, this article highlights the challenges arising from the current legislative trend of pursuing geopolitical and digital sovereignty within the framework of the internal market, particularly through reliance on Article 114 TFEU. It argues that this approach insufficiently captures the broader normative foundations of EU data governance. Consequently, the article advances an alternative perspective on the incremental evolution of CEDS, grounded in a reconceptualisation of solidarity, particularly external solidarity, understood as the pursuit of public interest objectives beyond the national and EU level.

#### 3.3.1 The notion of ‘solidarity’ in the context of international cooperation (external solidarity)

‘Solidarity’ is a key principle of EU law, reflecting the Union’s evolution from a purely economic organisation to a multi-dimensional entity with political, social, and human rights dimensions [30]. It is embedded in the Treaties and primarily operates as an internal organising principle, structuring cooperation between Member States and underpinning policy areas based on mutual support and shared responsibility.

The Court of Justice of the European Union has further reinforced its legal significance, recognising solidarity as a fundamental principle linked to the Union’s common interest and capable of justifying collective action, even where it may run counter to individual national interests [33,34].

Apart from being a principle constituting the imperative for internal solidarity, the treaties also touch upon its external dimension. In the preamble to TFEU, the EU confirms that ‘the solidarity principle binds Europe and the overseas countries’, going therefore beyond the creation of a specific bond between Member States – ‘desiring to ensure the development of their prosperity, in accordance with the principles of the Charter of the United Nations’ [35]. In general terms, Art. 21 TEU states that ‘The Union's action on the international scene shall be guided by the principles which have inspired its own creation, development and enlargement, and which it seeks to advance in the wider world. Principles of equality and solidarity are therefore also relevant in this context. More specifically, the Union’s external action should in that regard be ‘guided by unity and solidarity’, promoting partnerships and cooperation with third countries [14]. Such principles offer a more ethical and balanced framework for the harmonisation, liberalisation, and promotion of trade and investment in the context of international relations [36]. The deliberate incorporation of all external action objectives under the umbrella of Article 21 TEU was undertaken with the objective of enhancing the coherence and consistency of EU external relations [37].

#### 3.3.2 European health data space: between digital (Data) sovereignty and solidarity?

In the context of data sharing, a solidaristic approach is reflected in solidarity-based data governance (“data solidarity”), as described by the Digital Transformations For Health Lab [38]. The existing data governance schemes require rethinking, with the focus of data policies shifting from addressing data types to rather “emphasising evaluating data uses based on their potential and likelihood to generate public value” [39]. From the data solidarity perspective, the power asymmetries in data control within the digital ecosystem mean that data should not be viewed primarily as a matter of individual self-determination through personal control. Instead, the focus should shift toward collective control over data use, aiming to reshape existing dynamics by sharing both the benefits and risks more equitably across society.

Despite the transnational nature of CEDS and their emphasis on interoperability across sectoral data-sharing infrastructures, the strong emphasis on “digital (data) sovereignty” calls into question whether CEDS, in their present form, genuinely advance the idea of data solidarity. More specifically, do the governance models underpinning CEDS allow for collective societal oversight, as reflected upon in the concept of data solidarity? As van der Valk & Ryan [40] argues, “the values of the EU and the benefits for the common good of European society may get overlooked for the economic benefits of organisations if norms and social values are not considered..” While reflecting on the impact of data governance models in fostering data solidarity would certainly enrich this analysis, we intentionally leave it out of scope due to the methodological lens of this paper, as the breadth of the topic could merit a separate discussion.

The solidarity principle is especially relevant in the most sensitive sectors, where the potential usefulness of datasets not only goes beyond the narrow definition of health data - also encompassing data on factors influencing health - but also transcends national (and EU) borders due to the commonalities of issues faced (or potentially faced) by people worldwide. In this context, international cooperation that facilitates access to a greater volume of high-quality data - both health-related and concerning the broader factors that influence health - can contribute to more effective responses to common health threats (such as the COVID-19 pandemic), enable faster and more accurate diagnoses, and support the development of new treatment pathways. In an ideal scenario, this could help reduce social inequalities in access to healthcare.

The EHDSR provides a legal framework aiming to embed the concept of external solidarity, allowing third countries participation in the cross-border infrastructure for secondary use purposes (referred to as HealthData@EU). There are, however, several legal measures that these countries should meet to be connected to the infrastructure. According to Recital 80 EHDSR, third countries may become authorised participants in HealthData@EU, provided that the national contact point of a third country is compliant with the requirements in the Regulation. There is no explanation as to a practical meaning of this requirement, especially what is the interplay between this requirement with providing ‘access to health data users located in the Union, on equivalent terms and conditions’ (‘reciprocity’ principle). In practice, reciprocity stipulates that electronic health data should only be made available to a third country where the respective country allows access to its electronic health data by Union entities “under the same conditions and with the same safeguards as would be the case if they were accessing electronic health data within the Union” (Recital 94 EHDSR). The Commission will monitor the equivalence and, formally, the connection of a third country can only take place if the Commission establishes it by means of an implementing act (Art. 75 EHDSR).

Apart from necessary legal framework in place, Art. 75(6) EHDSR requires also a certain level of technical capability to connect to and participate in HealthData@EU. Moreover, third countries will have to comply with the requirements and technical specifications necessary to operate HealthData@EU and to allow them to connect to it (regulating secondary use of electronic health data), and reciprocal access rules. The Regulation seems to differentiate two scenarios: one concerning personal, and the other non-personal electronic health data sharing. Where the connection entails transfers of personal data related to the applicant or the health data user to third countries, relevant transfer instruments under Chapter V GDPR need to be in place for such transfers (Recital 81 EHDSR). It is, however, not clear whether this will be part of the implementing act, or there has to be an additional mechanism as laid down in Chapter V GDPR. Art. 75(5) EHDSR lists requirements for a third country to become an ‘authorised participant’, including compliance with Chapter V GDPR, but laying down the requirements necessary for an implementing act. The provision refers only to compliance with the requirements of HealthData@EU for the purposes of secondary use of health data, alongside requirement of compliance with Chapter IV EHDSR and the need to provide reciprocal access to health data users located in the Union to the electronic health data the authorised participants have access to. Art. 90 EHDSR further elaborates on additional conditions for transfer of personal electronic health data to a third country, leaving Member States a possibility to maintain or introduce further conditions on international access to, and transfer of, personal electronic health data. The limitations may be for instance, based on Art. 9(4) GDPR providing possibilities to limit the access to processing of genetic data, biometric data, or data concerning health. In that context, most of the categories listed in Art. 51 EHDSR would potentially fall under scope, offering Member States a possibility to considerably limit the scope of available data for third countries.

When it comes to non-personal data, the EHDSR underlines that, where such data is made available by HDABs to a health data user in a third country under a data permit, or through a health data request to authorised participants in a third country, and where the data is derived from a natural person’s electronic health data, it shall be deemed highly sensitive within the meaning of Article 5 (13) DGA, which lays down additional conditions for re-use. The term 'highly sensitive' is employed in two distinct contexts within the Union: firstly, in relation to public health, and secondly, in relation to public safety. Although these objectives are traditionally heavily Member State-centred, recognising non-personal health data as highly sensitive could open the possibility for the EU to frame more strategic objectives under public safety and health, for example when such data is leveraged in third countries to develop AI models or support innovation. At the same time, this raises the risk that economic benefits could be prioritised over key European values, and thus broader geopolitical, global, and solidarity-oriented dimensions of health data governance.

Although the EHDSR creates the impression that, once third countries are connected to the HealthData@EU infrastructure, they will become authorised participants on equal terms (as they must ultimately comply with the same requirements as EU Member States), Article 91 EHDSR reiterates the conditions applicable to health data applicants established in a third country. These include the requirements that (i) the third country be an authorised participant, and (ii) Union health data applicants are granted access to electronic health data in that country under conditions no more restrictive than those provided for in the Regulation. This reiterates the reciprocity requirement.

While the framework may foster international collaboration in the context of secondary use, the legal architecture for connecting third countries to HealthData@EU appears primarily aligned with economic and geopolitical objectives of developing own AI systems and digital infrastructures, rather than with external solidarity. In addition to the established requirement of compliance with Chapter V GDPR for international data transfers, third countries must comply with the governance and legal framework set by the EHDSR, achieve an appropriate level of technical capacity, and provide Union health data applicants with reciprocal access which, in current form, rather limits the solidarity, especially given that the reciprocity is strongly intertwined with the other requirements.

However, this approach risks discriminating against less technologically advanced countries, which may lack the necessary technical capabilities, as well as economically weaker countries, which may be unable to provide equivalent data-sharing conditions for EU health data users. Once again, the EHDSR has been observed to pursue ambitions aimed at consolidating the EU's digitally sovereign position on the international stage. These ambitions extend beyond the pure idea of harmonising the EU internal market, as foreseen by Article 114 TFEU, and overlook broader societal values based on solidarity principle, such as social justice and equal access to healthcare.

### 3.4 Intermediary conclusion: 114 TFEU overstretched?

Legislators have used Article 114 as a large legal base, overstretching the initial objective of the provision. In other words, legislators apply Article 114 TFEU to legislate matters directly connected to the internal market harmonisation, but also to regulate topics indirectly or disconnected to the internal market. Such movement turns this legal basis to a blank cheque to be used for EU legislators to adopt instruments in different topics, without important considerations that guide the block’s practices. This practice creates a general competence to the EU for adopting legal acts. Using this stretched interpretation of Article 114 allows for EU representatives to impose rules and values on both the Member States and their jurisdictions on various matters [41]. With this, Article 114 occupies a central piece to the discussion of possible different approaches to defend sovereignty.

Harmonising rules and establishing a solid internal European digital market became a major driver to reaching sovereignty goals, including technological sovereignty. However, sovereignty in itself is beyond the scope of Article 114. Without any specific definition of the term, the European Commission has used the idea of technological sovereignty to justify diverse policies in the Digital Agenda. Poli and Fahey presented an abstract definition to the term, showcasing how the EU’s main goal is to become completely independent in the management of technologies [42]. Following this rationale, the EU has adopted instruments that have a hidden goal of increasing and protecting sovereignty.

Applying Article 114 TFEU for hidden sovereignty ambition seems to be incompatible with certain ideas of sovereignty itself [43]. Amplification of its scope without considerations about other EU values and boundaries weakens the power of the legal basis. EU’s sovereignty must consider constitutional boundaries such as the need to protect human rights, democracy and the rule of law. Moreover, Articles 179 and 180 TFEU explicitly mention cooperation with third countries to ensure the strengthening of the EU's scientific and technological bases. Article 191 TFEU acknowledges the significance of collaboration with third countries in the context of safeguarding public health. Nevertheless, the opposite happened with the adoption of broad policies under the Article 114 umbrella. Part 4 will, therefore, present a hypothesis for regulating health data sharing in a way that is more aligned with societal and European values such as solidarity.

## 4 Reflections on Data Cosmopolitanism as A Normative Foundation of Global Health Data Space

In the most sensitive areas, such as healthcare, the central question concerns the vulnerable parties in the context of their respective relationships, such as patients and consumers. In that regard, data cosmopolitanism has been proposed as a theoretical response to health inequities as a global issue, one in which all communities share moral and political responsibility for the health of every person on the planet [12]. This approach to data governance is closely aligned with the idea of external solidarity, reflecting the need for cooperation beyond EU.

Data cosmopolitanism does not frame itself within geopolitical competition. Rather, it directs digital health innovation toward goals that are morally difficult to oppose, such as advancing health innovations resulting in better access to healthcare for everyone. Data cosmopolitanism rests on the idea of similarity, rooted in our shared humanity and the inherent dignity of every individual or community, regardless of variations in rights protections across systems. Moreover, data cosmopolitanism derives, in part, from states' obligations under international human rights law, including the duty to ensure the right to participate in and benefit from scientific progress, as well as the duty to cooperate in disease prevention. As Rueda et al [12] argue, broadening collaboration to a wider international community could yield “richer insights and representative results,” ultimately benefiting all. Moreover, since the costs of technological development are borne globally - particularly given the environmental impacts of building and maintaining AI systems (see, e.g., [44]), there is a strong moral imperative to extend the benefits of digitalisation equally. For these reasons, Part 4 puts forward the hypothesis of constructing a more globally oriented health data space in the future based on the principle of data cosmopolitanism. Furthermore, the hypothesis of a more global health data space guided by data cosmopolitanism assumes that Member States can uphold their international human rights obligations in the health sector, and that the EU regulates in accordance with the principle of solidarity enshrined in Article 21 of the TEU. In this framework, data cosmopolitanism would also entail a more consistent inclusion of third countries within the secondary use of health data infrastructure.

In the ensuing sections, the paper will first analyse and evaluate how the current, predominantly internal market-based approach within the EHDSR does not promote data cosmopolitanism. This analysis will highlight the shortcomings of the existing EHDS regulation and provide the building blocks and argument for the suggested hypothesis (Section 4.1). Subsequent reflections on alternative mechanisms for article 114 TFEU, are proposed as a valuable point of departure for future research into the establishment of a hypothesised global health data space. This alternative legal basis aims to strike a balance between the health data sovereignty of Member States and their obligations under human rights law, ultimately fostering shared objectives of promoting equity in global healthcare (Section 4.2).

### 4.1 Pitfalls of current approach of EHDS in light of data cosmopolitanism

Rueda et al [12] contrast data cosmopolitanism with data nationalism and regionalism, which prioritise national or regional interests in data governance. Data cosmopolitanism seeks to elevate the global community, extending principles such as justice, solidarity, and cooperation beyond regional boundaries. Central to this approach is reciprocity, requiring states and international actors to mutually recognise and uphold obligations, enabling trust and cooperation in electronic health data sharing beyond national or regional domains.

Reciprocity encompasses mutuality and proportionality. Mutuality ensures that obligations correspond across parties, while proportionality balances the benefits and burdens borne by each actor [45]. Legal equality - freedom to consent and to assert the same rights for oneself as for others - remains essential, even if practical equality among states varies [45,46].

Internationally, this is reflected in Principle 7 of the Rio Declaration on Environment and Development, which emphasises cooperation “to conserve, protect and restore the health and integrity of the Earth's ecosystem” [47]. The principle recognises differences in contributions to global degradation, calling for “common but differentiated responsibilities,” with developed countries bearing greater responsibilities due to societal pressures, technology, and financial resources [47].

In contrast, the EHDS builds on lessons from the COVID-19 pandemic, where coordinated EU-level responses facilitated secondary use of health data. However, this solidarity remains internally focused: HealthData@EU enables data sharing across Member States, with only limited provisions for third countries. EHDSR enforces technical and governance standards uniformly, including for bilateral arrangements with non-EU partners, privileging equivalence of obligations defined by EU rules rather than accommodating differences in resources or capacity among global actors.

While data cosmopolitanism calls for inclusive, proportionate, and globally reciprocal frameworks, EHDSR’s reliance on Article 114 TFEU prioritises internal market harmonisation and EU-centric solidarity. Consequently, when combined with the technical and governance requirements imposed by other EU instruments, such as the DA and the DGA, as discussed in Section 3.2, there is a risk that an “EU model” of data governance will be effectively exported beyond the Union. In practice, this model may privilege actors that are technologically advanced or economically aligned with the EU, while limiting the participation of third countries and constraining the development of more globally inclusive, solidarity-based approaches. This dynamic is reminiscent of the so-called Brussels effect [19].

At the same time, alternative approaches, especially those grounded in the concept of data cosmopolitanism, retain significant normative and practical value, especially in fields such as health, where global cooperation and data sharing are essential.

Since EHDSR imposes standards on all Member States, even bilateral cooperation with third countries will likely follow the same (European digital sovereignty) requirements. Reciprocity as a fundamental component of EU solidarity with third countries does not demand identical obligations, but equivalence of value reflecting the balance of benefits and burdens. In relationships between countries with unequal power, nations with fewer resources should contribute proportionately to their capacity, consistent with the principle of reciprocity.

Thus, there is a structural mismatch between the cosmopolitan idea - global justice in health and data use - and the EU’s legal competence under Article 114 TFEU, focused on internal market harmonisation and sovereignty. Extending EHDSR to third countries is foreclosed, as the legal focus remains anchored in European (internal) solidarity. In practice, this tends to exclude less-resourced countries rather than fostering broader inclusion. Article 114 demonstrates a clear preference for market-making over humanitarian solidarity, producing a normative tension with the global cosmopolitan hypothesis that has its relevance in the domain of health.

### 4.2 Legal pathways for establishing a global health data space beyond article 114 TFEU

As demonstrated in Section D of Part 3, the argument that the imposition of cooperation in the domain of health via an EU way of data governance within Data Spaces is derived from the scope of internal market policy, and brings about undisclosed digital sovereignty objectives, can be subject the subject of scrutiny. Moreover, given the absence of broad goals of harmonisation and the potential for national legislation to diverge, which is specific to the domain of health, the EHDS highlights the ongoing need for a delicate balancing act between the sovereignty of Member States and the EU's (implied) regulatory powers in specific domains. In addition, national health data access bodies within the EHDS retain a pivotal function in the granting and regulation of data permits for subsequent data usage. This approach is again predicated on the premise of preserving state sovereignty, a principle that is manifested through the allowance of member states to exercise control over access to and use of data [48]. As demonstrated by Article 168(7) TFEU, Member States retain the prerogative to formulate their own (global) health policies and to organise and deliver health services, as well as to manage them and allocate the resources allocated to them. The introduction of digital sovereignty objectives and requirements under the guise of the internal market in this regard restricts and contradicts the abilities of Member States.

In conclusion, the paper recommends further research to establish a more balanced legal foundation for the EU's health regulation and external and foreign actions in this domain. This recommendation is made in light of accommodating diverging state sovereign approaches towards healthcare and enabling Member States to abide by international human law obligations in the domain of health as well as for the EU to act according to the principle of solidarity in its international actions. The theory of data cosmopolitanism provides a more globalised, balanced (based on reciprocity) and solidarity-based viewpoint to tackle health data sharing in Data Spaces in the future.

A significant question that has yet to be addressed is the precise nature of the competence of the EU to regulate the exchange electronic health data for secondary use, and whether the EU could simply assume decision-making powers based on other grounds than 114 TFEU in this area. The selection of appropriate legal bases for regulatory actions is typically undertaken by the Commission, Parliament and Council. Selecting an incorrect option, or one which does not align with the objectives of the regulatory framework, could be grounds for annulment of the regulatory action by the CJEU. Consequently, in order to strengthen the hypothesis of data cosmopolitanism in the construction and regulation of future health data sharing and Data Spaces, an appropriate legal foundation that accommodates this theory must be selected.

With regard to this particular issue, the EU has incorporated a measure of flexibility or residual powers into its treaties in instances where no specific competence norm is present. The purpose of this incorporation is to enable the achievement of its objectives in these areas that are characterised by a multitude of interwoven objectives, as is the case in the EHDS. The concept of residual or implied powers is predicated on the premise that such powers are derived from either explicitly conferred powers or the objectives of the EU itself [49]. The criteria of unanimity or necessity, as outlined in Articles 352 TFEU, represent the principle of subsidiarity and can thus only serve as a legal foundation for the establishment of an international agreement if the Treaties lack the requisite authority to do so and if the goals cannot be sufficiently achieved by Member States alone (here with regard to the establishment of an EHDS) [50]. Consequently, with respect to global Data Spaces, the viability of internal market objectives or undisclosed digital sovereignty objectives is questionable in meeting this necessity and subsidiarity test. National traditions must be given due consideration, and the establishment of a European power to act externally must be achieved prior to any further developments.

Accordingly, Article 352 TFEU enables the EU to adopt suitable measures in instances where it lacks explicit competence (for instance, health domain) [49]. For instance, prior to the Treaty of Maastricht and 168 TFEU, the legal and policy framework governing health was largely shaped by Article 352 TFEU [51]. In this regard, Article 352 TFEU represents a continuation of the principles of conferral and subsidiarity. Article 352 TFEU establishes a procedure for the adoption of regulatory instruments that requires the unanimous agreement (contrary to 114 TFEU that requires a qualified majority) of all Member States [52]. The requirement for unanimity as opposed to a qualified majority in the vote has the potential to ensure the legitimacy of broader EU powers is achieved in consultation with the sovereignty of Member States [53]. These arguments underscore the potential of Article 352 TFEU for the regulation of health by the EU, as well as for the legitimisation of the EU's external actions in this domain by Member States. Nevertheless, while 352 TFEU is a feasible undertaking, it remains underused in practice. Consequently, further research is required in this area to clarify the grey zones in specific regulatory areas or matters [54].

Another instrument that is of interest in the context of the EHDS is that of Article 216 (1) TFEU. In accordance with the stipulations outlined in this Article, the EU may enter into an agreement with one or more third countries or international organisations, provided that the Treaties permit such an action or if the conclusion of an agreement is deemed necessary to achieve one of the objectives referred to in the Treaties [55]. The legal effects of such an agreement would be binding on the Member States. However, the argument that internal market objectives should prevail over other health-related considerations, as well as over relationships with third countries, would be difficult to sustain in practice under the necessity test set out in Article 216 (1) TFEU. In this respect, Article 216 (1) TFEU has the potential to support a more balanced approach, accommodating principles such as data cosmopolitanism and reciprocity. By requiring that external agreements be necessary for the exercise of EU competences, it may ensure that relations with third countries or international organisations remain sensitive to Member States’ competences and national traditions, or to powers that have not been conferred upon the Union.

In this context, it has been argued that the advantages accruing from EU external action for Member States outweigh the disadvantages associated with the use of implied powers under Article 216 (1) TFEU. This serves as an example of how Article 216 can translate the ideas brought by the data cosmopolitanism theory, especially in cases where global cooperation is aligned with reciprocal national health interests. By applying Article 216 (1) TFEU as a legal basis for data policies, the EU could better translate the potential of international cooperation to achieve shared goals. Instead of weaking the rationale of digital sovereignty, Article 216 (1) TFEU could serve as an instrument to ensure EU's participation in global initiatives for common values such as solidarity, without disharmonising positions among the Member States.

With this, the paper concludes with the recommendation that further research be conducted into Article 352 TFEU as an alternative legal basis for Article 114 TFEU and 216 (1) TFEU for potential international agreements that are binding on Member States to fulfil the hypothesis of a more global health data space, building on the concept of data cosmopolitanism. In this respect, the selection of Article 352 or 216 (1) TFEU over Article 114 TFEU facilitates disapproval or a more balanced approach with regard to the enforcement of internal market and digital sovereignty objectives on a pan-European scale, under the pretext of the internal market. In addition, regarding data cosmopolitanism, Articles 352 and 216 (1) depart from and ensure that Member States can honour their own international human rights law and the principles of conferral and subsidiarity prevail.

## 5 Conclusion

The EU has demonstrated its ambition to play a leading role in establishing global standards and creating an environment that supports data-driven solutions and technological development. CEDS such as the EHDS are the tangible implementation of this novel EU data governance ambition. However, when formulating legislative frameworks and policies relating to the EHDS based on the classical legal basis of Article 114 TFEU for regulating the internal market, the EU also pursues narratives of strategic autonomy - the aim to reduce dependence on non-EU actors in critical economic and technological domains - and digital sovereignty - the objective of controlling and protecting data flows, infrastructure, and digital technologies within the Union. This paper has shown that regulating under 114 TFEU gives rise to 'hybrid' policies, which encompass classic normative powers for developing an internal market as well as geopolitical aspirations and protectionist measures. This is also demonstrated by holding the EU Data Strategy, and CEDS policies in particular, against the principle of solidarity. There is a tendency for EU data sovereignty ambitions to exceed the scope of establishing an internal market as stipulated in Article 114 TFEU, thereby limiting the potential for non-EU countries to participate in the EU data-sharing ecosystem.

In relation to the tensions inherent in the specific use case of the EHDS and the domain of health typically characterised by national traditions, the authors propose the hypothesis of data cosmopolitanism to look at EHDS as a potential global health data space. Data cosmopolitanism does not constitute the sole means through which the evolution of a prospective health data domain can be assessed; nonetheless, this theoretical framework offers a compelling perspective when confronted with the problematic issues that have been delineated in this article. After all, when acting on a more international level and speaking about non-EU countries, the EU's sovereignty approaches are not balanced with other European principles, such as the principle of solidarity. This is particularly relevant in relation to Member State sovereignty and their need to honour their international human rights law obligations in terms of health. The hypothesis of data cosmopolitanism is proposed to achieve a more balanced approach in this regard. The exploration of alternative legal bases, other than Article 114 TFEU, for the regulation of the health domain while accommodating the hypothesis of data cosmopolitanism holds considerable potential for future research. Consequently, Article 352 or 216 (1) TFEU should be considered as a potentially fruitful point of departure for further research in this area.

## Limitations

A key limitation of this paper lies in the choice of the EHDS as a case study for exploring the potential of creating a legal framework to enable international cooperation in data sharing. Given the structural and developmental differences among CEDS, as well as their diverse establishment methods and organisational forms, insights derived from the governance of health data may not be fully transferable to other sectors. Consequently, while our analysis may inspire further developments in data governance within the forthcoming Data Union Strategy, the sector-specific features of each data space must be carefully considered.

Furthermore, this paper relies heavily on the novel concept of data cosmopolitanism as developed by Rueda *et al*. Although we build upon these findings, the conceptual framework of data cosmopolitanism still requires further clarification and research—particularly in relation to other sensitive sectors where international cooperation is critical, such as environmental protection and energy.

## Ethics Statement

The authors confirm that they have read and comply with the ethical requirements for publication in Data in Brief. Furthermore, the current work does not involve human subjects, animal experiments, or any data collected from social media platforms.

## Credit Author Statement

**Marta Musidlowska:** Conceptualisation, Methodology, Formal Analysis, Writing - Original Draft, Supervision. **Michiel Fierens:** Conceptualisation, Methodology, Formal Analysis, Writing - Original Draft. **Isabela Maria Rosal:** Conceptualisation, Methodology, Writing - Original Draft.

## Declaration of Generative AI and AI-assisted Technologies in the Writing Process

During the preparation of this work the author(s) used ChatGPT 4o to improve language and readability. Perplexity.ai was also used to do quick searches of the relevant legislative texts and to format the bibliographic references. After using these tools/services, the author(s) reviewed and edited the content as needed and take(s) full responsibility for the content of the publication.

## Footnotes

1 After the Lisbon Treaty, the TFEU consolidated the adoption of “internal market” as the unique concept to represent this definition. Before, there were discussions if internal and common market would represent different concepts. In this paper and considering the harmonising text adopted by the TFEU, internal and common market are used as synonyms.
